# Microglia–Astrocyte Cooperation and Peripheral T Cells in Alzheimer’s Disease: State-of-the-Art and Treatment Perspectives

**DOI:** 10.3390/ijms27073295

**Published:** 2026-04-05

**Authors:** Giulia Bivona, Giulio Ghersi

**Affiliations:** 1 Department of Biomedicine, Neurosciences and Advanced Diagnostics, University of Palermo, 90127 Palermo, Italy; 2 Department of Biological, Chemical and Pharmaceutical Sciences and Technologies (STEBICEF), University of Palermo, 90128 Palermo, Italy; giulio.ghersi@unipa.it

**Keywords:** Alzheimer’s diseases, neuroinflammation, immune response, lymphocytes, microglia, treatment strategies

## Abstract

Alzheimer’s disease (AD) is a neurodegenerative disorder first described more than one century ago. Over this time, many features of the disease have been discovered and, consequently, many different approaches in the diagnosis and treatment of AD have been developed. A major assumption has guided research on AD in the past: this fatal form of cognitive decline is believed to have a pathogenic basis in the deposition of amyloid beta (Aβ) aggregates throughout the brain. Consequently, a main goal of AD therapy is to reduce Aβ load, and several monoclonal antibodies targeting amyloid are among the most recent approaches to AD treatment. However, the effectiveness of these drugs is limited, as they cannot block the progression of the disease; they only slow it down in certain conditions. Many other causative factors are known to promote the development of the disease, with immune system involvement being the most investigated. Indeed, it has been well documented that the microglial response enhances the deposition of other altered proteins, such as Tau, and induces a neurotoxic microenvironment that promotes neuronal loss. In this scenario, the interaction between microglia and astrocytes is known to accelerate pathogenic processes, and a possible role for peripheral T lymphocytes in AD pathology has also been described. An interesting hypothesis is that immune cells driving chronic inflammation might worsen AD progression and, therefore, could represent a target for treatment strategies in this disease. Thus, this review article aims to summarise the role of brain and peripheral immune molecules and cells in AD. Also, immune-based treatments for AD are described, including those targeting microglia and T cells.

## 1. Introduction

AD is a neurodegenerative disease with complex pathogenesis, involving protein aggregation and neuroinflammation. Two proteins, Aβ and Tau, are known so far to form aggregates and deposits within the brain during AD. The presence of these altered proteins (redundant, misfolded, or hyperphosphorylated) in the intracellular and extracellular spaces leads to neuronal death, which, in turn, causes cognitive and behavioural decline in AD patients. However, some other factors could be responsible for the complex biochemical, pathophysiological, and histochemical features of AD, including inflammation and the adaptive immune response [1,2,3,4].

Microglia, the resident innate immune cells of the brain, have been shown to link Aβ aggregation to the deposition of hyperphosphorylated Tau [5], driving fatal AD neuropathologic phenomena (intracellular Tau deposition, disseminated inflammation throughout the brain, and neuronal depletion [6,7]).

Along with brain-resident cells, peripheral immune cells are known to participate in AD, and many studies have investigated the role of adaptive immunity in AD, both in animal and in vivo models [8,9,10]. Attempts to treat AD by modulating the adaptive response are described.

Innate and peripheral immune cells’ responses are also linked to oxidative stress, which largely contributes to neuroinflammation. Oxidative stress results from an imbalance between reactive oxygen and nitrogen species, normally generated by metabolic processes, and the endogenous antioxidant systems that protect the body from oxidative damage [11]. Oxidative stress influences the brain environment, driving neuroinflammation through several mechanisms: it activates inflammatory signalling pathways and enhances the production of damage-associated molecular patterns (DAMPs) and senescent cells, thereby eliciting innate immune cell reactivity [12]. Also, oxidative-stress-induced microglial activation initiates an inflammatory cycle that drives the production of additional reactive oxygen species [13]. Although oxidative stress is considered a key factor driving AD pathology, the efficacy of antioxidant drugs to treat AD has gained controversial results, with promising findings achieved in animal models alongside disappointing data in clinical trials [14]. Nonetheless, it remains a valuable target for novel AD treatments, especially in the early stages of the disease [14].

Although advanced knowledge in the field of AD has been achieved in the recent two decades, some questions remain to be addressed: (i) the question regarding the pathophysiology of the disease, with particular attention to the role of inflammation; (ii) the question around easy-to-perform, blood biomarker testing for diagnosis; and (iii) another question around effective, disease-modifying treatments, focusing on the chance to develop drugs targeting immune cells and immune molecules.

Innate and adaptive immune cells have been documented to interact during AD, and the evidence that lymphocytes infiltrate the brain, worsening AD pathology, has been provided [10]. Understanding the coordinated interactions between glial and adaptive immune cells is important because it may offer a valuable approach for identifying novel molecular targets for developing AD drugs. Indeed, modulating neuroinflammation has emerged from experimental studies as a good opportunity to attenuate AD progression.

This review article briefly reports the role of brain and peripheral immune molecules and cell types in AD, and summarises immune-cell-based therapeutic approaches. The literature was searched based on language, relevance, and publication year using the following keywords: “microglia”; “reactive”; “astrocytes”; “Alzheimer’s disease”; “drugs”; “T lymphocytes”.

## 2. Alzheimer’s Disease

AD is a neurodegenerative disease responsible for most cases of dementia, and it affects almost 50 million people worldwide [15]. Because of the extended average lifespan, the number of AD cases is expected to triple over the next three decades [16]. Due to its high mortality rate and high health burden, it represents a fatal social and economic concern. AD can exist in two forms: the most common sporadic form, with ageing as its major risk factor, and the familial form, which is present in APOEɛ4 carriers. However, the disease is thought to have many other risk factors, including environmental variables and genetics [17]. Clinically, AD comes with neurobehavioural and cognitive symptoms, compromising memory and language abilities, together with affective dysfunctions. AD patients also lose autonomy in daily life activities. Neuronal loss is the main factor underlying these disturbances. AD histopathological hallmarks include Aβ extracellular deposits and intracellular hyperphosphorylated Tau aggregates. The membrane protease enzyme beta-site amyloid precursor protein (APP)-cleaving enzyme 1 (BACE1) and the gamma secretase enzyme generate extracellular Aβ deposits by cleaving APP, producing various toxic amyloid peptides. Due to one-to-one binding, Aβ peptides form oligomers, polymers, and plaques, with oligomers probably being the more neurotoxic of the three [18,19]. Tau protein is normally part of neuronal microtubules, but it can undergo some alterations, like hyperphosphorylation, leading to aggregates or tangles.

From the diagnostic perspective, the National Institute on Ageing and the Alzheimer’s Association Working Group (NIA-AA) guidelines and the Amyloid, Tau, and Neurodegeneration (ATN) framework [20,21] established that cerebrospinal fluid (CSF) biomarkers can help diagnose AD, along with the imaging tools and clinical observation. Main CSF biomarkers include Aβ40, Aβ42, the Aβ42/Aβ40 ratio, pTau 181, and tTau, and their measurement in blood is expected to become part of routine examination in AD patients. Biomarkers have been considered a valuable tool for predicting the progression of the disease across the so-called AD continuum, which refers to the evolution from a biological entity defined by biomarkers to a clinical entity defined by symptomatology [22]. However, it should be noted that whether biomarkers can predict progression to AD from the objective cognitive decline is a debated matter [23].

Other biomarkers linked to the molecular mechanisms underlying AD are currently under investigation. Among these, inflammatory molecules are of particular interest, having recently been included in the ATN framework [24]. Indeed, it is widely accepted that Aβ deposition triggers inflammation in the brain, and increasing evidence indicates that resident innate immune cells, such as activated microglia, play a fundamental role in the onset of the disease [6]. Besides resident immune cells, peripheral immune molecules and cell types have been documented to accompany the progression of AD [25], with particular attention gained by lymphocytes.

The search for AD biomarkers is also justified by the need to identify molecular targets for disease-modifying treatments, as the AD therapeutic approach appears to be improving. Immunotherapeutic strategies for AD are currently based on monoclonal antibodies targeting Aβ, although these drugs still present some limitations [26] (see Section 5 on “Treatment Perspectives: Immune-Derived Molecular Targets for Novel Drugs”).

## 3. Microglia- and Astrocyte-Mediated Neuroinflammation in AD

Microglia are brain-resident innate immune cells that perform many functions, including defence against pathogens and maintenance of brain homeostasis. Due to their highly active movements, microglia can continuously contact other brain cells, including neurons and astrocytes, to sense damage and injury arising from the nervous microenvironment [27,28]. Once activated by a stimulus, microglia change their phenotype to clear damage and restore homeostasis. Several stimuli are known to promote changes in microglia, including DNA, Adenosine triphosphate (ATP), abnormal protein aggregates like amyloid, and pathogens. Resting microglia lack phagocytic ability and are unable to clear Aβ deposits. In response to various insults, microglia become reactive, and numerous reactive phenotypes have been described. Among different microglial phenotypes, disease-associated microglia (DAM) are activated and have been associated with AD [6]. DAM display increased phagocytosis and endocytosis, along with cytokine overproduction. The DAM phenotype, also known as the neurodegenerative microglial phenotype, surrounds amyloid aggregates and is thought to contribute to disease progression [29]. Recently, it has been demonstrated that the deletion of the immune-checkpoint molecule TIM-3 gene Havcr2 in microglia is responsible for their shift toward the DAM phenotype, shedding light on the role of immune checkpoints in microglia-mediated mechanisms underlying AD [30].

**Figure 1:**
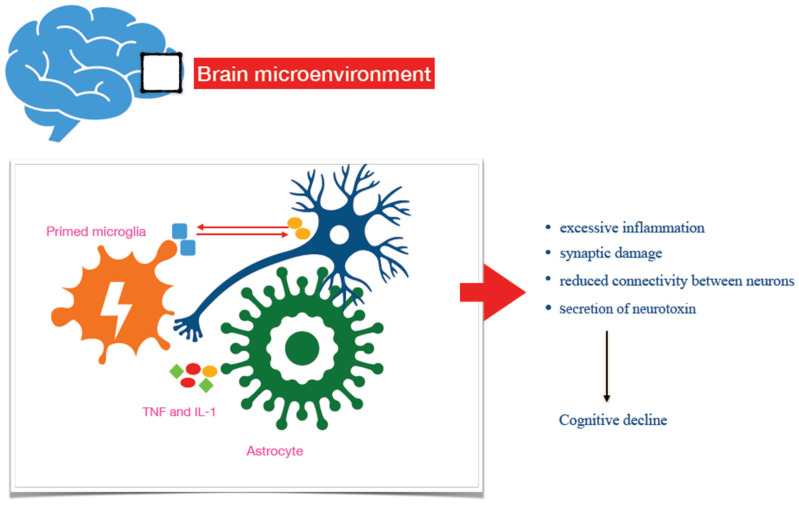
Microglia–astrocyte and microglia–neuron crosstalk drives AD pathogenic features, such as excessive inflammation and neuronal dysfunction. Neuron-to-microglia crosstalk normally maintains brain homeostasis. Dysfunctional neuron-to-microglia interplay causes chronic activation and priming of microglia, with over-secretion of pro-inflammatory cytokines. Tumor Necrosis Factor (TNF), Interleukin 1 IL-1, and the complement component 1q (C1q), secreted by microglia, activate the NF-kB signalling pathway in astrocytes, promoting their A1-like polarisation. A1-like reactive astrocytes exert neurotoxic actions and kill neurons directly through a soluble toxin.

While transient activation of microglia leads to neuroprotective and anti-inflammatory effects in the brain, chronic activation drives neurotoxicity and is thought to contribute to the onset and progression of neurodegenerative diseases [6,31] (Figure 1). Indeed, repeated stimuli (such as Aβ aggregate deposition) turn microglia toward a primed phenotype, which displays detrimental features, including the loss of neuroprotective functions, inability to resolve inflammation, and a massive release of pro-inflammatory cytokines [32]. The primed microglial phenotype drives chronic neuroinflammation, which, in turn, renders the brain more prone to neuronal damage and loss, promoting neurodegeneration [6]. Consistently, the aggressive, pro-inflammatory phenotype of microglia can be considered a major target for developing novel tools to approach AD pathophysiology and therapy [33,34]. Specifically, factors interfering with the activation and priming of microglia—including neuronal and microglial molecular products (C-X3-C Motif Chemokine Ligand 1, CX3CL1 and triggering receptor expressed on myeloid cells 2, TREM2)—have been regarded as potential targets for novel treatment strategies of the disease (Table 1), with pilot studies addressing their potential usefulness in AD therapeutic approaches [35,36,37]. Also, artificial intelligence-based therapeutic strategies targeting the interplay between inflammatory and neuronal cells could represent a valuable opportunity for treating AD (for a detailed review of this topic, see ref. [38]).

Astrocytes are glial cells supporting several brain functions, including neurotransmission, maintenance of the integrity of the blood–brain barrier, synaptic pruning, and survival of neurons through the secretion of neurotrophic factors [39]. Upon stimulation, astrocytes undergo polarisation toward distinct reactive phenotypes, and several reactive states are known, characterised by changes in their functional and molecular patterns. However, all the different subsets can be classified into two major categories based on the features they exhibit, namely neuroprotective or neurotoxic [40]. A1-like reactive astrocytes display neurotoxic, pro-inflammatory characteristics, while A2-like polarised subsets show neuroprotective and proliferative functions [41]. Polarisation toward A1 strictly depends on the microglia, with microglia–astrocyte crosstalk necessary for astrocytes to mount an inflammatory response upon stimulus. The secretion of interleukins and complement components by microglia induces the activation of the nuclear factor kB (NF-kB) signalling pathway, which, in turn, promotes the up-regulation of pro-inflammatory genes in astrocytes and modifies their transcriptional activity [41,42]. A1-like reactive astrocytes exert neurotoxic effects, including the down-regulation of synaptogenic signals, leading to synaptic damage and reduced neuronal connectivity [43]. Also, they up-regulate complement components and produce chemotactic factors, enhancing the pro-inflammatory milieu within the brain [44]. Further, A1-like reactive astrocytes kill neurons directly through the secretion of a soluble toxin [42]. It has been documented that the healthy brain contains 20% A1-like astrocytes, whereas in neurodegenerative disorders, 60% reactive astrocytes can be detected [42]. Indeed, in situ hybridisation and immunofluorescence studies have reported a high presence of A1-like astrocytes within disease-associated brain regions, as measured by the colocalisation of normal and reactive astrocyte markers (Table 1). Finally, the down-regulation of the astrocyte-associated molecule clusterin can drive alterations in phosphorylated Tau mediated by microglia and reduce synapse numbers, increasing susceptibility to AD (for more details, see [45]).

Microglia–astrocyte crosstalk is relevant to AD pathology, and molecules driving such interplay might represent valuable targets for an AD therapeutic approach [46].

## 4. Adaptive Peripheral Immune Cells in the Pathophysiology of AD

Lymphocytes are immune cells belonging to the adaptive arm of immunity that respond to antigens to fight pathogens. Two main types of lymphocytes are known, according to their roles in the cytotoxic and humoral immune responses—T- and B-lymphocytes, respectively. Although a possible role for CD19+ CD20+ B cells in the pathophysiology and treatment of AD [47] has been investigated, T cells have attracted greater attention. The role of T cells in AD is yet not fully elucidated; nonetheless, data on their role in the pathophysiology of the disease are strong and numerous. Collectively, this evidence has been considered a solid background for hypothesising a concrete translational impact of novel AD treatment strategies (see Section 5 on “Treatment Perspectives: Immune-Derived Molecular Targets for Novel Drugs”).

T lymphocytes are divided into three major groups: CD8+ T (Tc) cytotoxic ones, killing infected cells; CD4+ T Helper (Th) cytotypes, helping other immune cells to exert their functions; and regulatory T lymphocytes (Tregs), having a major role in counterbalancing immune response to avoid adverse immune reactions.

T cells have the ability to rearrange their T-cell receptor (TCR) genes to bind specific antigens derived from pathogens. The possible repertoire of TCR sequences is very extensive, which hampers the complete profiling of specific T cells and the understanding of their role during certain immune-related diseases [48]. However, new techniques for identifying antigen-specific T lymphocytes have enabled the discovery of disease-associated T cells in AD, and age-related CD8+ T cells have been detected [4,49]. Since age-related CD8+ T lymphocytes could mediate pathogenic mechanisms of AD, these data shed new light on the mechanism underlying the onset and progression of this neurodegenerative disorder [49]. New technologies in this field include RNA/TCR sequencing, 3D structural modelling of TCRs, protein-structure prediction networks, mass cytometry, Major Histocompatibility Complex (MHC) peptidomes, and the use of integrated bioinformatic tools.

Altered, excessive, or chronic T-cell responses lead to uncontrolled inflammatory reactions. Since chronic inflammation in the brain is a major feature of AD, it has been suggested that a dysfunctional T-cell response could enhance neuroinflammation and sustain the pathophysiology of the disease [50]. Furthermore, a complex interplay among microglia, astrocytes, and T cells has been reported, forming a loop that exacerbates the pathogenic mechanisms of AD [10,51].

A possible role for T lymphocytes in the onset and progression of AD has been posited since high blood levels of pro-inflammatory cytokines in AD patients were reported [25,52]. Also, a role for lymphocytes in the pathophysiology of AD has been hypothesised following studies in animal models [53,54,55,56]. Generally, main conclusions from all the studies performed are the following: (i) during AD, T cells migrate from the periphery to the brain, with many the possible mechanisms by which they could be recruited within the central nervous system [50,57,58,59]; (ii) once T cells enter the brain, they actively influence the neuroinflammatory microenvironment by cooperating with other immune cells [10,51,57]; (iii) during AD, T cells can be either protective or harmful toward the brain environment, based on two main variables—shifting in new subsets, and AD clinical stages [57].

Among T cells, CD4+ Th lymphocytes have been extensively studied for a long time, and they are regarded as a major contributor to the pathophysiology of AD. CD4+ Th lymphocytes are classified based on the cytokines they produce, and can display pro-inflammatory (Th1 and Th17) or anti-inflammatory (Th2) properties. Th1 and Th17 cells have been shown to infiltrate the brain during AD, contributing to worsening the pro-inflammatory milieu through the production of their cytokines. Th17 lymphocytes have gained particular attention, both because IL-17 levels have been found to be increased in AD patients [60] and because blocking IL-17 has been shown to reduce cognitive symptoms in rats [50,61]. Studies in animal models demonstrated increased serum and CSF interleukin 17 (IL-17) after injection of Aβ42 [62]. It has been suggested that IL-17 could be involved in the development and progression of AD by acting as an attractant for neutrophils, facilitating their recruitment and functions [63]. Also, IL-17 has been proposed to interfere with microglial-mediated Aβ phagocytosis [64].

CD8+ Tc lymphocytes have been considered a relatively homogeneous cell type, despite recent findings suggesting that they could be classified into different subsets [65]. The discovery of an expanded subpopulation of CD8+ Tc lymphocytes—CD8+ T effector memory CD45RA+ (TEMRA) cells—in the blood and CSF of AD patients opened the way for the hypotheses that the adaptive immune response is actively involved in neurodegenerative processes, with T cells playing a major role [59,66,67]. However, a full understanding of the role of different CD8+ Tc subtypes and their influence on AD onset and progression is lacking.

There is general agreement on the protective role Treg cells could play in AD [68]. These cells have been documented to reduce the pro-inflammatory load in the neuro-microenvironment by various mechanisms, including inhibiting the release of pro-inflammatory cytokines within the brain and attenuating astrocyte responses [48]. Also, circulating Tregs are sharply reduced in AD patients compared with healthy subjects [69]. Drugs enhancing Tregs have been developed, including rapamycin and low-dose IL2, although their use is not extended to AD [57,70].

## 5. Treatment Perspectives: Immune-Derived Molecular Targets for Novel Drugs

AD has been considered a hopeless disorder for a long time due to the lack of effective, disease-modifying therapies. Recently, breakthroughs in monoclonal antibody development targeting Aβ have partially changed AD treatment. However, many steps forward are needed to find drugs that block the progression of the disease and limit side effects. Some hope lies in new molecular targets within the biochemical pathways regulating the interactions among microglia, adaptive immune cells, and neurons (Table 1). Producing an anti-inflammatory microenvironment and avoiding disseminated inflammation and neuronal loss after Aβ aggregate deposition are the major functions required of new targets.

## 6. Immunotherapy Against Aβ

Several monoclonal antibodies against Aβ are available, with differences as to the epitope of amyloid that is used as the molecular target of the drug [71]. Although differences in antibody efficacy have been reported, they share a common objective: reducing Aβ load. Such ability has been shown to vary across different molecules, as defined by positron-emission tomography (Aβ PET), with a correlation between Aβ load reduction and slowing of disease progression [72,73,74]. Unfortunately, some limitations affect current immunotherapy against Aβ. Firstly, clinical trials using these drugs exhibit heterogeneity in terms of patients recruited and treatment features, including administration doses and timing [71]. Secondly, monoclonal antibodies against Aβ load can be relatively effective only at low Aβ load, suggesting that late-stage AD remains a fatal disease with very limited slowing of disease progression [75]. Theoretically, biomarkers could help identify the disease in the pre-symptomatic stage, allowing for early treatment with immunotherapy targeting Aβ; however, there is no consensus on the usefulness of biomarkers as predictive tools for monitoring the disease [23]. Further, in the hypothesis of biochemical screening for AD prevention, there is an urgent need for easy-to-perform, low-cost, and standardised blood biomarkers.

Exploratory studies on several molecules involved in the biochemical pathways of innate immune cells are in progress. Peripheral (cytokines) and central molecules have been identified as potential targets for intervention. For instance, small molecules that reduce the release of pro-inflammatory cytokines by inhibiting the NF-kB pathway have been shown to attenuate neuroinflammation in preclinical models of neurodegeneration, such as PD [14]. Among the central targets, immune molecules produced by neurons and microglia—such as CX3CL1 and its receptor—have attracted attention for their ability to modulate the duration of the innate immune response [27].

The NOD-like receptor pyrin domain-containing 3 (NLRP3) inflammasome is responsible for the secretion of pro-inflammatory cytokines, making it a potential target for AD drugs; however, NLRP3 inhibitors have shown some side effects in preclinical models, limiting their therapeutic potential [76].

Other molecules, including immune checkpoints and T-cell-derived products, are under investigation (for more details on these targets, see refs. [77,78]).

Overall, microglia-related molecules such as TREM2 and T-cell-derived therapies have attracted the most attention.

### 6.1. Microglia-Derived Molecular Targets

TREM2 is a receptor protein common to some cell types, including microglia. Initial interest in TREM2 has arisen from genome-wide association studies (GWAS), reporting that variants in this gene are associated with a higher risk of developing AD [79]. In microglia, TREM2 modulates inflammation by reducing pro-inflammatory cytokine secretion and shifting the homeostatic microglial phenotype toward a disease-associated microglial phenotype, which represents a primed subset [80,81]. Homeostatic microglia, even activated, are able to counterbalance their pro-inflammatory neurotoxic features (that are necessary to remove Aβ by phagocytosis) with anti-inflammatory, neuroprotective characteristics (which are essential to limit neuroinflammation, maintaining an optimal microenvironment in the brain) [6]. In contrast, primed microglia display an aggressive phenotype, lacking the capacity to resolve inflammation, leading to detrimental effects. Indeed, primed microglia are associated with neurodegeneration and AD. Exploratory studies to develop TREM2-based drugs for AD use agonist antibodies targeting specific epitopes of the protein, enhancing the TREM2 signal [78]. Although a few promising results have been reported, agonising TREM2 to treat AD is a controversial strategy because excessive microglial activation induced by TREM2 can fail to promote amyloid removal and even exacerbate Tau pathology [82]. Further, the contribution of TREM2 agonism to AD appears to depend on many other variables, such as genetic variants in the protein and disease stage [83].

### 6.2. T-Cell-Derived Molecules

In 2024, the “T Cells in the Brain” symposium took place at Columbia University, and multiple opportunities for the use of T-lymphocyte-related drugs in AD were proposed [4]. One intriguing example is the use of an anti-CD3 antibody to activate peripheral Tregs and modulate microglial phenotypes [84]. The antibody, to be administered intranasally, offers the following advantages: it does not cross the blood–brain barrier, and it is non-invasive. The basic assumption underlying this molecule’s testing is that stimulation of Tregs (albeit indirect) and modulation of microglia attenuate inflammation in the brain [84]. The study has been performed in a 3xTg mouse model, after testing the antibody in immune-mediated diseases such as lupus, diabetes, and arthritis [84]. In 2025, a case of a 78-year-old moderate AD patient treated for three months with the anti-CD3 antibody was reported. The case report showed beneficial effects of the antibody, including a reduction in the uptake of the 18 kDa translocator protein ligand targeting microglia [85], as measured by 18F-Florbetapir-PET (18F-PBR06-PET). Apparently, this constitutes preliminary data and interest in this molecule is prudent; however, an ongoing early clinical study on nasal anti-CD3 in neurological diseases has started [85].

Among T-cell therapies for AD, it is worth mentioning antiviral treatments and their related trials. It is known that some infections have been linked to the development of AD, especially those caused by pathogens with tropism for the nervous system. These include herpes simplex virus type 1 (HSV-1), human herpesvirus (HHV), Chlamydia pneumoniae, and some parasites [86], but most of the evidence concerns HSV-1 [87]. An altered immune response due to infection, along with chronic inflammation and disruption of the blood–brain barrier, is the most probable mechanism by which AD begins after infections [4]. Early clinical trials have been performed on the effect of antiviral Valacyclovir on mild cognitive impairment and AD [88,89]. Recent findings on the use of antiviral medicines in AD also include transposable elements (TEs), and exploratory studies are ongoing. TEs are DNA sequences that vary in their position within the genome [90]. They can be activated by viral infections, leading to altered or dysfunctional sequences that promote the development of neurodegenerative diseases [91]. The dysregulation of AD-associated TEs could be modified by using valacyclovir, with possible translational application on the incidence of the disease [92].

## 7. Limitations

This narrative review presents some limitations—including the lack of human data, the paucity of data on the role of T lymphocytes in AD, and the limited evidence for microglia-targeted drugs in humans—due to the shortage of available studies on this topic.

## 8. Conclusions

Immunotherapies targeting Aβ have become more effective in recent years. However, therapies blocking disease progression and improving the quality of life in late-stage AD patients are still lacking. Although the possible roles of innate and adaptive immune responses in the development of AD might open a new avenue in the search for treatment strategies for the disease, the use of drugs targeting neuroinflammation and immune responses, including non-steroidal anti-inflammatory drugs (NSAIDs) and TREM2-based drugs, has not been supported by evidence so far. Nonetheless, new technologies available in this era suggest that innovative therapeutic approaches for AD could be based on manipulating these cell types and their networks. In particular, modulating microglia subsets, targeting the complex interplay between microglia and astrocytes, and guiding the selection of engineered TCRs could represent a new landscape for possible AD second-generation treatments.

## Figures and Tables

**Table 1:** Immune-derived therapies for neurodegenerative diseases.

Molecule	Diseases	Target	Mechanism	Molecule Type
Foralumab	AD, PD	Microglia, T cells	Modulation of microglia activation; enhancement of Treg function	Monoclonal antibody
Bapineuzumab, Solarezumab, Crenezumab, Gantenerumab, Aducanumab, Lecanemab, Docanemab	AD	Aβ	Recognising Aβ epitopes, removing Aβ deposition, and lowering Aβ load	Monoclonal antibody against Aβ
Low-dose IL-2	PD, ALS	Tregs	Expansion of Tregs and enhancement of their regulatory function	Cytokine
Antibody AL002	AD	Microglia, T cells	Activation of microglia; enhancement of Treg function	Monoclonal antibody
Rapamycin	PD, ALS	mTOR kinase	Enhancement of their regulatory function; reduction in CD4+ T-cell activation; impairment of innate immune response	Macrolide
RRx-001, VTX2735	PD, ALS	NLRP3 Inflammasome	Inhibition of pro-inflammatory cytokines IL-1β and IL-18; suppression of inflammation and immune response	NLRP3 inhibitors

PD: Parkinson’s disease; ALS: Amyotrophic Lateral Sclerosis; AD: Alzheimer’s disease; Aβ: amyloid beta; mTOR: mammalian target of rapamycin; NLRP3: NOD-like receptor pyrin domain-containing 3.

## Data Availability

All data supporting opinions and conclusions reported in the current manuscript are available at PubMed.com.
